# Monodispersed CsPb_2_Br_5_@SiO_2_ Core–Shell Nanoparticles as Luminescent Labels for Biosensing

**DOI:** 10.1021/acsanm.0c03340

**Published:** 2021-02-12

**Authors:** Cynthia Collantes, Victoria González Pedro, María-José Bañuls, Ángel Maquieira

**Affiliations:** † Instituto Interuniversitario de Investigación de Reconocimiento Molecular y Desarrollo Tecnológico (IDM), Universitat Politècnica de València-Universitat de València, Camino de Vera s/n, E46022 València, Spain; ‡ Departamento de Química, Universitat Politècnica de València, Camino de Vera s/n, E46022 València, Spain

**Keywords:** cesium lead bromide nanocrystals, silica growth, CsPb_2_Br_5_@SiO_2_ core−shell nanoparticles, monodisperse, stability, luminescent label, protein detection

## Abstract

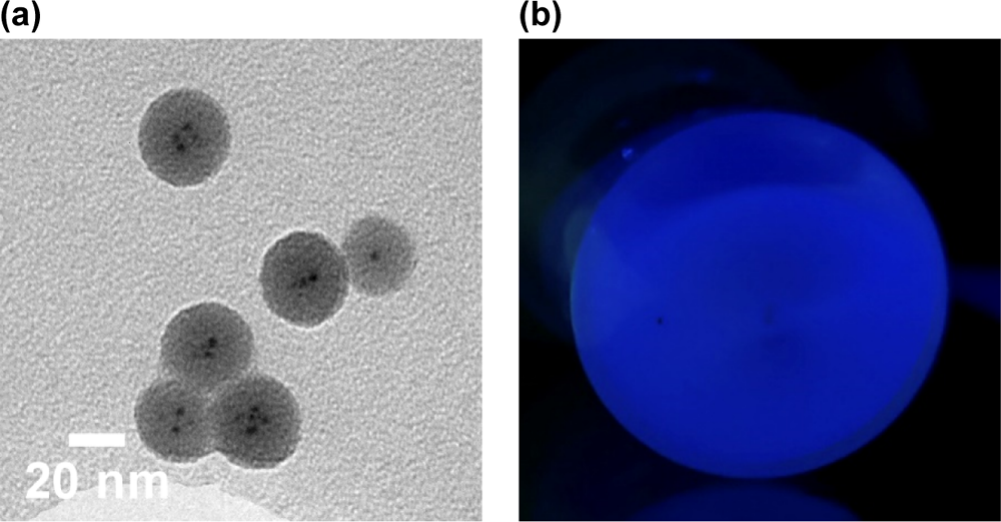

Despite the rising advances in the field of metal halide perovskite nanocrystals (NCs), the exploitation of such nanoparticles as luminescent labels for *ex vivo* imaging and biosensing is still unclear and in the early stages of investigation. One of the major challenges toward the implementation of metal halide perovskite NCs in biosensing applications is to produce monodispersed nanoparticles with desired surface characteristics and compatible with aqueous environments. Here, we report the synthesis of monodispersed spherical CsPb_2_Br_5_@SiO_2_ core–shell nanoparticles by post-synthetic chemical transformation of 3D CsPbBr_3_ NCs in the presence of tetraethyl orthosilicate and a critical water/ammonia ratio. This method involves an ammonia-mediated and ammonia-induced “top-down” transformation of as-synthesized 3D CsPbBr_3_ NCs to smaller CsPb2Br5 nanoclusters (ca. 2–3 nm), which trigger a seed-mediated silica growth, yielding monodispersed spherical blue luminescent (λ_emission_ = 432 nm) CsPb_2_Br_5_@SiO_2_ perovskite nanoparticles. By adjusting the reaction conditions, core–shell nanoparticles of a 36.1 ± 4.5 nm diameter, which preserve their optical properties in water, were obtained. Besides that, the viability of the developed nanoparticles as a luminescent label for biosensing has been proven by specific biorecognition of the IgG protein in a direct immunoassay. Our work sheds light on the chemical processes and transformations involved in the silica nucleation mechanism in the presence of perovskite nanoparticles and opens the way for the future rational design of the next generation of semiconductor NC luminescent biological labels.

## Introduction

Metal halide perovskite (MHP) nanocrystals (NCs) have attracted great interest owing to their excellent optical features such as composition-tunable band gap, easy synthesis, and high photoluminescence quantum yields (PLQYs = 50–90%) and have demonstrated superior properties in photovoltaics and light emission devices.^1^ Besides that, they also possess higher-order nonlinear optical properties,^2−4^ with reported absorption cross sections for up to five photons,^4^ which make them a promising luminophore with a bright future in many areas of modern biology such as labels and contrast agents for *in vitro* bioimaging and biosensing (i.e., immunostaining, DNA and protein microarraying, and flow-cytometry-based diagnosis) and for super-resolution imaging applications going beyond the optical diffraction limits.^5^

Toward their implementation as luminescent probes, is necessary NCs confinement inside suitable protective materials which accomplishes a dual role: prevent their decomposition in a water medium and endow their chemical functionalization with appropriated groups. In this framework, the use of alcoxysilanes is a smart and very straightforward route for encapsulating MHP NPs because silica is chemically stable and transparent in the whole visible region, protects materials against moisture-induced damage, and possesses facile conjugation with different functional groups to enable further functionalization.^6^

Accordingly, some successful examples have been reported.^7−20^ However, most efforts using these conventional SiO_2_ coating methods for perovskite NPs failed because perovskite NCs are too sensitive to conditions of Stöber methodology (i.e., the presence of water in a basic medium), which leads to their dissolution.^21^ This involves working in soft conditions, resulting in random alkoxysilane polymerization processes. Thus, until date, most of the reported methods were only successful in dealing with an ensemble of MHP NCs and the products were macroscale particles. In this sense, gaining insight into the silica growth mechanism on MHP and the development of a methodology to synthesize monodispersed MHP@SiO_2_ are very challenging tasks and only few works have reported their encapsulation into an oxide matrix at a single-particle level. Some examples have successfully achieved this goal by adaptation of sol–gel processes. For instance, Cheng et al. reported room-temperature crystal growth of CsPbBr_3_ inside pre-synthesized amine-functionalized silica micelles. ^19^Ding et al.^20^ developed a reverse microemulsion methodology employing *n*-hexylalcohol: cyclohexane mixture, ammonium hydroxide, and triton *x*-100 as surfactant, and Hu et al.^17^ combined a water-triggered transformation process of Cs_4_PbBr_6_ NCs to CsPbBr_3_ and a sol–gel method for preparing CsPbBr_3_@SiO_2_ and CsPbBr_3_@Ta_2_O_5_ Janus NPs. On the other hand, other researchers have focused on alternative methods for SiO_2_ coating. Among them, Huang et al.^11^ report *in situ* growth into hollow siliceous nanospheres and Song et al.^9^ synthesized perovskite core–shell nanocubes via a modified hot injection method, wherein a certain part of oleylamine was substituted with (3-aminopropyl)triethoxysilane (APTES).

Here, we report a novel and effective approach for preparation of monodispersed silica NPs containing MHP NCs based on the controlled chemical transformation of 3D CsPbBr_3_ in the presence of tetraethyl orthosilicate and a critical water/ammonia ratio. Under these reaction conditions, 3D CsPbBr_3_ NCs transform into CsPb_2_Br_5_ nanoclusters of 2–3 nm, which act as silica nucleation seeds rendering monodispersed spherical blue luminescent CsPb_2_Br_5_@SiO_2_ perovskite nanoparticles (NPs). The facile and effective functionalization of the developed nanoparticles with vinyl and amine groups by easy post-synthetic treatment was confirmed by attenuated total reflectance Fourier-transform infrared spectroscopy (ATR–FTIR).

Besides that, in this study, the developed NCs were used as a fluorescent label in immunoassays for specific detection of bovine serum albumin (BSA). For that purpose, we developed the perovskite NCs labeled antiBSA antibodies, which were applied to the specific biorecognition of proteins. BSA was used as an example of a target protein.

## Results and Discussion

In our methodology, colloidal CsPbBr_3_ NCs (11.04 ± 2.19 nm edge length, PLQY ∼ 90%) with an emission maximum at 515 nm were synthesized according to published protocols.^22^ (See Figure S1 in the Supporting Information for more details). Then, for synthesizing silica-overcoated MHP NCs, 0.5 mL of CsPbBr_3_ (40 nM in toluene) was treated with 2 μL of concentrated aqueous ammonia (25% w/w) and different tetraethylorthosilicate (TEOS) equivalents. The mixture was incubated overnight under stirring (300 rpm) and the resulting MHP@SiO_2_ core–shell NPs were collected by centrifugation and dispersed in toluene. The content of NH_4_^+^ is critical in the shell growth and formation of isolated core–shell nanoparticles because ammonia serves as a basic catalyst for the hydrolysis/condensation process of the silica precursor, reduces the amount of energy to star nucleation, and also has a role in protecting the newly formed silica particles from aggregation.^23^ In our work, we optimized the ammonia content to 2 μL of a concentrated base. Larger volumes produce a degradation of perovskite nanoparticles, while lower volumes lead to a random nucleation of TEOS monomers in a macroscale assemble.

The concentration of TEOS and water play a key role in the spatially controlled deposition of SiO_2_. First, to evaluate the influence of TEOS concentration, we analyzed the morphology of the silica shell (grown on the NPs surface) at different alkoxysilane equivalents and a constant volume of water (2 μL of concentrated aqueous ammonia 25% w/w). Structural features and reactant concentrations for the resulting SiO_2_-overcoated perovskite nanocomposites are summarized in Table S1.

**Figure 1:**
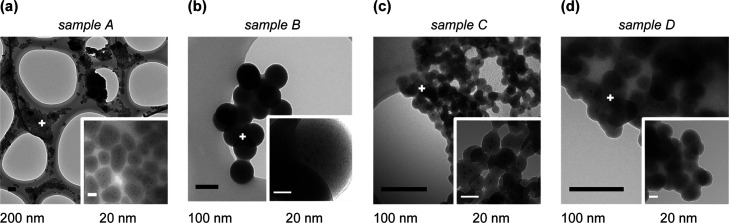
TEM images of MHP@SiO_2_ core–shell nanocomposites prepared with increasing concentrations of TEOS (a–d).

Figure 1 shows the transmission electron microscopy (TEM) images of samples A–D treated with different amounts of TEOS (60, 310, 480, and 960 μL, respectively). It is remarkable that all TEM images present 2–3 nm perovskite NCs embedded into silica nanoparticles with different sizes and morphologies. Sample A shows MHP NCs encapsulated into ellipsoidal silica nanoparticles with a major diameter and a minor diameter of 35.5 ± 8.4 and 20.7 ± 3.4 nm, respectively. In addition, it also exhibits a small population of nanoparticles (162 ± 38 nm-averaged diameter) and nanowires (56 ± 14 nm-thick), which could be attributed to the ripening mechanism and rearrangement of original CsPbBr_3_ NCs into larger structures.^24−26^ In sample B, the resulting nanomaterial evolved into the formation of spherical silica nanoparticles of 101.4 ± 6.5 nm (average diameter). However, samples C and D render a silica nanoparticle cross-linked network with an average diameter of 19.7 ± 2.4 and 33.8 ± 4.0 nm, respectively.

Remarkably, larger amounts of TEOS volumes led to the formation of macroscale ensembles (Figure S2a). This fact is attributed to the large amount of silane, which produces random polymerization of alkoxysilane monomers in macroscale composites. These data are similar to those obtained for the control sample in the absence of basic aqueous equivalents (Figure S2b), which points out the key role of the aqueous ammonia in the nanoparticle nucleation mechanisms.

Another critical parameter controlling the silica shell growth is the water amount. To test the influence of water in shell morphology, 0.5 mL of CsPbBr_3_ NCs was treated with 310 μL of TEOS, 2 μL of aqueous ammonia, and increasing water volumes. As perovskite particles decomposed in the excess of basic aqueous medium, a fractioned dropped method was adopted with the aim to slow down this process, in which TEOS and ammonia dissolved in water were added with a flow rate of 0.74 and 0.1 μL min^–1^, respectively.

**Figure 2:**
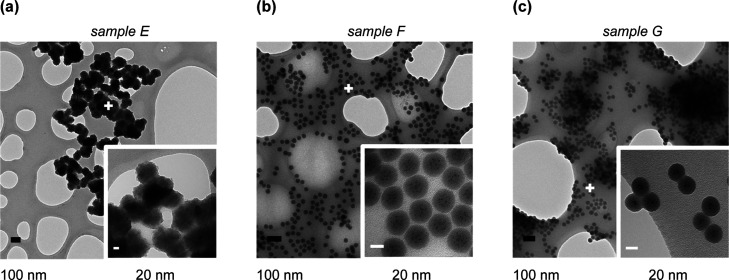
TEM images of MHP@SiO_2_ core–shell nanocomposites prepared with increasing concentrations of water (a–c).

As shown in Figure 2 and Table S1, monodispersed core–shell nanoparticles with an average diameter size in the range of 30 nm were achieved for samples F and G treated with 28 and 48 μL of water, respectively. Larger volumes of water produce hydration of CsPbBr_3_ NCs, triggering their decomposition up to their ionic precursors,^27−29^ therefore leading to the formation of core-free silica nanoparticles (Figure S3). The differences observed between samples A and E prepared with the same reagent concentration arise from the dropped fractioned methodology adapted for preparation of sample E. These data highlight the key importance of water in obtaining highly monodispersed nanoparticles. In addition, the experiment was repeated, but this time increasing the final volume of the TEOS reagent to 480 and 960 μL. In both cases, the resulting products led to silica agglomeration (see Figure S4, Supporting Information).

In order to understand these observations, we first need to consider the chemical processes involved in the nucleation of silica: hydrolysis and polycondensation of TEOS. With less equivalents of water, most of the TEOS monomers will remain unhydrolyzed; thus, the process is driven by the spontaneous polycondensation of TEOS alkoxide species, which leads to a random nucleation mechanism, resulting in size and shape variability of silica nanoparticles (as shown in samples A to E). With 28 and 48 μL of water, 30 and 50% of TEOS monomers are partially hydrolyzed. Since these monomers are deprotonated due to the high pH value, in the presence of MHP NCs, hydrolyzed TEOS monomers are attracted toward Pb atoms and hence replace the original capping molecules, leading to a localized growth of uniform-sized silica shells.

**Figure 3:**
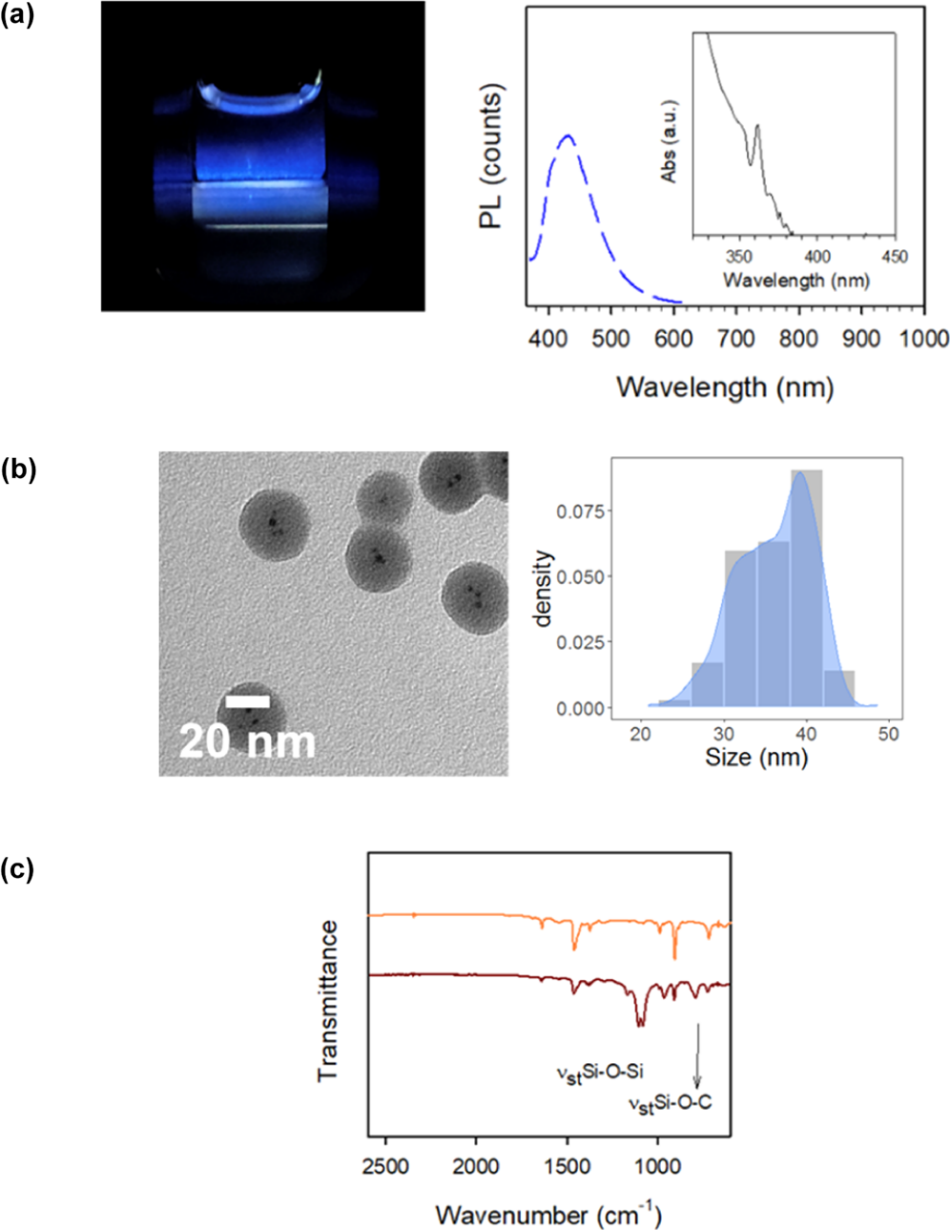
(a) Image of blue-emitting core–shell MHP@SiO_2_ NCs at λ_exc_ = 254 nm. Absorbance (black line) and emission spectra (blue line) of MHP@SiO_2_ NCs. The PL spectrum was recorded at an excitation wavelength λ_exc_ = 355 nm. (b) TEM image of monodispersed core–shell nanoparticles and particle characterization. (c) ATR–FTIR spectra of reference MHP NCs (orange line) and MHP@SiO_2_ core–shell NCs (brown line).

The structural and optical features of monodisperse core–shell nanoparticles from sample G are summarized in Figure 3a,b and Table S2. The as-synthesized nanoparticles exhibit a blue luminescent peak centered at 432 nm with a PLQY of ∼ 5% and an average particle diameter of 36.1 ± 4.5 nm.

Paying attention to the existing literature, although there is a variety of works which agree on structure and composition of CsPb_2_Br_5_ NCs, they greatly diverge on the interpretation of their intrinsic optical properties, which currently is a subject under debate. For instance, there are works which attribute not noticeable photoluminescence (PL) to CsPb_2_Br_5_ NCs.^30,31^ Other works describe CsPb_2_Br_5_ clusters with an UV emission edge at 380 nm^32^ and a dual emission behavior (∼460 and 520 nm) with an emission quantum yield of 4%.^33^ Besides that, other reports on CsPb_2_Br_5_ nanoplatelets and microplates suggest a material with strong blue to green tunable emission properties.^34−36^ Unfortunately, the exact electronic structure and PL mechanism in CsPb_2_Br_5_ is not completely known and the emission properties have yet to be fully established because of the existence of direct and indirect transitions.^30,37^ Although this topic is worthy to be studied, we considered that is beyond the scope of this manuscript.

The ATR–FTIR spectra of bare and core–shell NCs are shown in Figure 3c. The peak at ∼1050 cm^–1^ corresponds to the asymmetric stretching of Si–O–Si linkage and confirms the formation of a cross-linked siloxane network surrounding the perovskite nanoparticle. There is also a peak at 790 cm^–1^ that corresponds to the Si–O–C symmetric stretching of the unreacted alkoxysilane groups. EDX–TEM elemental analysis (Figure S5) reveals that the atomic Si/O ratio of the shell was 1:2, close to the theoretical value.

In order to identify the nanoparticle core composition and determine the Cs, Pb, and Br stoichiometric element relations, inductively coupled plasma mass spectrometry (ICP–MS) measurements were carried out. The elemental analysis results are shown in Table S3 in the Supporting Information. Paying attention to the Cs-to-Pb obtained ratio (0.56) reveals the formation of CsPb_2_Br_5_ perovskite-related NCs. Concerning the large Br-to-Pb molar ratios registered, they can be attributed to the technical difficulty for determining bromide species by ICP–MS, which typically interferes with the Ar dimer of plasmogen gas.^38−40^

**Figure 4:**
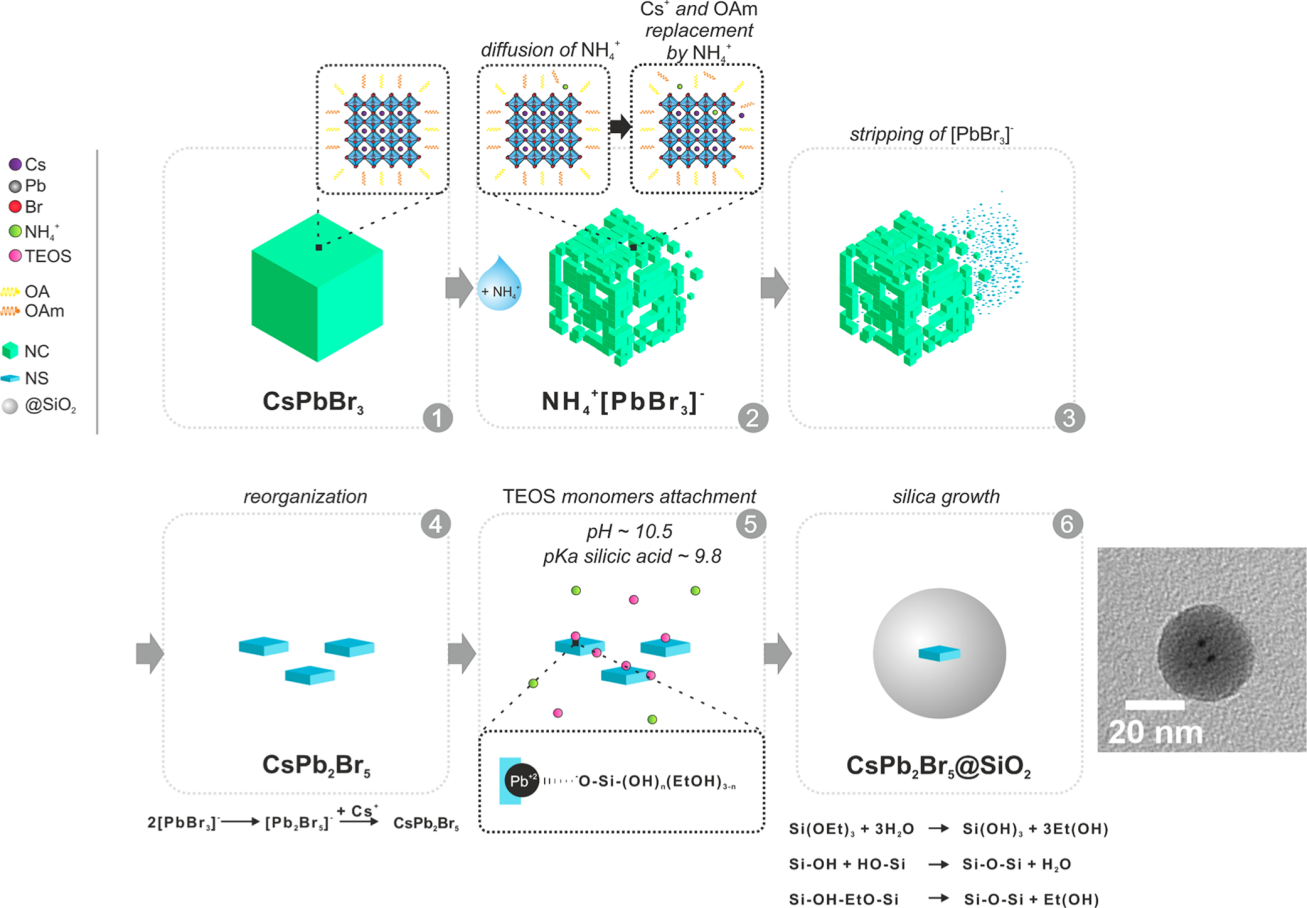
Schematic representation of the formation mechanism of CsPb_2_Br_5_ silica-coated core–shell NPs from CsPbBr_3_ NCs. The proposed mechanism involves diffusion and intercalation of NH_4_^+^ cations into a CsPbBr_3_ native structure (step 1–2), exfoliation of [PbBr_3_]^−^ smaller nanoclusters through a toluene–water interface (step 3), reorganization into CsPb_2_Br_5_ NCs (step 4), and seed-mediated growth of uniform silica shells (steps 5 and 6).

On the basis of the results discussed above, we suggest that the transformation process is probably controlled by the intercalation of ammonia cations, the replacement of Cs positions, and the variation of the coordination number of Pb^2+^ in water. A plausible schematic mechanism of the crystal structure evolution is shown in Figure 4 and eqs 1 and 2, and the related processes can be described as follows. The first step is the diffusion of ammonia in the perovskite network; then, Cs^+^ cations in the perovskite material are replaced by NH_4_^+^ to form NH_4_PbBr_3_, resulting in distinct changes in the crystalline structure. This fact, along with the replacement of oleylamine by ammonia molecules, induces exfoliation of *x*NH_4_^+^[PbBr_3_]^−^ smaller nanoclusters through the toluene–water interface, which will interact to form CsPb_2_Br_5_ NCs (see eqs 1 and 2). It is noteworthy that a recent work of Liu et al. reports the change of the coordination number of Pb^2+^ from six to eight and transformation to CsPb_2_Br_5_ in a humid environment.^41^ In another work, Balakrishnan and Kamat^33^ reported phase transformation under dodecyldimethylammonium bromide treatment. Although in this latter phase, transition only occurs for long alkyl chain ammonium cations, our work differs because of the combined effect of water and ammonium. Then, deprotonated TEOS monomers can attach to lead dangling bonds of the released NCs, triggering a seed-mediated growth of uniform silica overcoating surrounding the perovskite NCs and blocking the formation of larger CsPb_2_Br_5_ nanosheets.
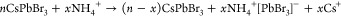
1
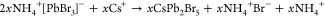
2

In addition, high-resolution transmission electron microscopy (HR-TEM) measurements were also carried out with the aim to unveil the nature of perovskite NCs. However, it has not been possible to determine the crystal lattice using HR-TEM either because of the small proportion of the crystal lattice or because of the considerable SiO_2_ shell thickness, which make its detection difficult.

In an additional experiment, increasing the reaction time from 24 to 48 h, the formation of agglomerated perovskite core–shell NCs was observed. Among them, there was a small population of silica nanoparticles which contain larger perovskite NCs (∼7.5 nm) embedded inside. Probably, the presence of these NCs is due to the formation of larger NCs from etched Cs^+^ and NH_4_^+^[PbBr_3_]^−^ at longer reaction times, which are then encapsulated inside silica shells. The HR-TEM image of these NC cores reveals that the interfringe distance is about 0.3 nm (Figure S6), corresponding to the (220) lattice plane of the crystal,^33,36^ thus confirming the formation of CsPb_2_Br_5_ NCs.

The absence of core-free silica nanoparticles in sample G could be explained according to LaMer theory,^42,43^ which claims that the energy barrier governing the heterogeneous nucleation is lower than that governing the homogeneous nucleation, and empty nanoparticles only form when the concentration of TEOS monomers surpasses the homogeneous nucleation threshold. In this sense, our fractionated drop method can always meet the above-mentioned conditions because fresh TEOS is added after the previous TEOS is mainly consumed.

The stability of synthesized core–shell nanoparticles has been confirmed by dispersing 5 mg of the collected NCs in 5 mL of water and sonicating for 2 min (Sonorex Super, Bandelin Co., Germany) with a frequency of 50/60 Hz. As shown in Figure S7, the blue emission of the core–shell nanostructures could still be observed clearly after 4320 min (3 days).

**Figure 5:**
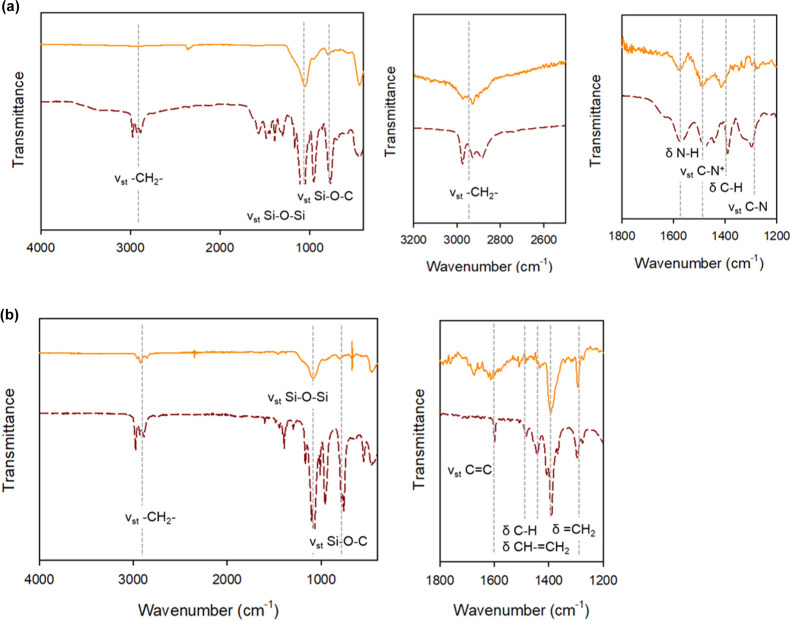
FTIR spectra of reference alcoxysilanes (brown line) and MHP@SiO_2_ core–shell NCs (orange line) functionalized with (a) 3-aminopropyltriethoxysilane and (b) vinyltriethoxysilane.

The easy and effective functionalization of the developed nanoparticles with amine- and vinyl-functional groups was demonstrated by additional post-synthetic treatment with alkoxysilanes bearing the respective terminations and confirmed by ATR–FTIR spectroscopy (Figure 5). NCs functionalized with APTES present characteristic stretching of Si–O–Si (1050 cm^–1^) and Si–O–C (780 cm^–1^) and the strong asymmetric and symmetric stretching vibration modes of CH_2_ at 2923 and 2854 cm^–1^, respectively. They also present −NH (1579 cm^–1^), C–N (1490 cm^–1^), and C–N (1315 cm^–1^) bands, which confirms the presence of APTES on the surface of perovskite NCs.^44^ The absence of the NH_2_ stretching band (3300–3400 cm^–1^) could be attributed to hydrogen bindings that result in a peak broadening in the infrared spectrum of a molecule. On the other hand, core–shell nanoparticles functionalized with vinyltriethoxysilane exhibit the characteristic features of the vinyl group (i.e., ν_st_ (C=C): 1629 cm^–1^; δ (CH_2_=CH):1503 cm^–1^; δ (=CH_2_): 1382 cm^–1^).^45^ These results support the versatility of our NCs, whose surface chemistry could be easily adjusted by post-synthetic treatment with adequate alkoxysilane.

**Figure 6:**
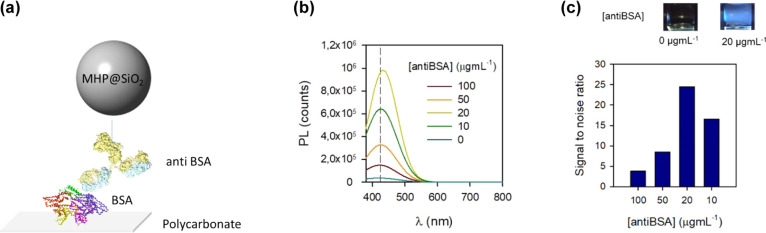
(a) Direct immunoassay scheme. (b,c) PL response and signal to noise ratio obtained for different NP/antibody ratios. The top image in (c) depicts a picture of samples treated with 0 and 20 μg mL^–1^ of anti-BSA.

Finally, as a proof of concept, the capability for immunosensing of fluorogenic labels based on the developed MHP NCs conjugated to the IgG antibody has been proven by a direct immunoassay onto polycarbonate cuvettes, Figure 6a. For this purpose, core–shell NPs were conjugated to the anti-BSA antibody by passive adsorption. In parallel, polycarbonate (PC) cuvettes were incubated overnight with the BSA protein in buffer saline phosphate (PBS, pH = 7). After washing, the NP–anti-BSA antibody conjugate was added in cuvettes and incubated for 2 h. Finally, PL was recorded (for further experimental details see the Supporting Information).

In Figure 6b,c are depicted the PL response and signal to noise ratio for cuvettes treated with anti-BSA–NP conjugates at different concentrations. It is noteworthy that these results confirm the successful biorecognition of the target, achieving a signal to noise ratio of 25. Moreover, a control experiment where BSA-coated cuvettes were treated with NPs in the absence of antibodies presents a negligible PL response. The lower signal to noise ratio registered for samples treated with a higher antibody concentration could be attributed to the large antibody/nanoparticle ratio, which may lead to a high antibody loading and a reduction of the immunoreactivity because of the steric hindrance.^46,47^ Although additional research needs to be done, such as the attainment of MHP core–shell NCs that preserves their native PL yield and the optimization of antibody/nanoparticle ratio, our work goes one step forward and demonstrates the feasibility and potential of perovskite nanoparticles as a labeling tool in immunochemistry.

A comparison of our work with the current state of the art is depicted in Table S4 (Supporting Information). Remarkably, there are several works which report the encapsulation of CsPbBr_3_ NCs into an oxide matrix at a single-particle level. Considering the requirements of luminescent nanoparticles as a fluorescent label, nanoparticles with an average diameter between 1 and 100 nm present dimensions comparable to biological functional units and have been demonstrated to be effective for biosensing and bioimaging applications.^48^ In addition, they should be also monodisperse, stable in an aqueous medium, and preferably spherical because of the more efficient immobilization of biomolecules on the nanoparticle surface without variations in curvature.^49^ Among the different works in the literature, there are only three reports that accomplish all requirements. On the one hand, Zhong et al. described quasi-spherical CsPbBr_3_@SiO_2_ core–shell NPs prepared via a supersaturated recrystallization method in which perovskite precursors were injected into a bad solvent (toluene) containing the alkoxysilane solution.^18^ Song et al.^9^ synthesized core–shell cube-shaped nanoparticles via a modified hot injection method, wherein oleylamine was partially substituted with APTES and Huang et al. prepared core–shell NPs via *in situ* growth into a hollow siliceous nanosphere template.^11^

In this sense, our work constitutes an alternative approach to the existing field for controlled synthesis of monodispersed core–shell NCs with suitable properties to be used as a fluorogenic label based on the post-synthetic treatment of as-synthesized CsPbBr_3_ NCs with TEOS and aqueous ammonia. This work sheds light on the chemical processes involved in the silica nucleation mechanism in the presence of perovskite nanoparticles and the role of ammonia and fractioned water addition in the phase transformation and the formation of spherical and monodisperse CsPb_2_Br_5_@SiO_2_ core–shell NPs. Although we are fully aware that the emission efficiency of our developed NPs is too low and further investigation is mandatory to prepare NPs which maintain their native structure and PLQY intact, for instance, the study of a larger basic catalyst, which does not penetrate into a crystal network or the introduction of pre-passivation steps of CsPbBr_3_ NCs.

With respect to applications, Song et al.^9^ and Ding et al.^20^ employed their developed NPs for cell imaging in tumoral culture. In this sense, our work makes the difference because we develop a fluorescent specific label via the formation of antibody-conjugated nanoparticles for specific protein detection. Thus, our developed NPs can combine the small size and benefits of perovskite nanoparticles with the abilities of antibodies for specific recognition of a selected target. An accurate analysis of biomarker molecules is essential for the early detection, treatment, and management of diseases. This implies applications for diagnosis by immunoassay in cell or immunostaining or their use as a multimodal fluorescent contrast agent in cell tracking, transfection, and so forth.

## Conclusions

In summary, in this work we have demonstrated the synthesis of spherical blue luminescent monodisperse CsPb_2_Br_5_@SiO_2_ core–shell NPs, fluorescent labels for biosensing, via a ligand-mediated transformation of pre-synthesized CsPbBr_3_ NCs in the presence of ammonia, water, and TEOS. The easy and effective functionalization of the developed nanoparticles with amine and vinyl groups by additional post-synthetic treatment with corresponding alkoxysilanes was confirmed by ATR–FTIR spectroscopy. Finally, going one step further, we demonstrated the application of the developed core–shell NCs as luminescent for biosensing by QD-labeled anti-BSA antibody fluorescence immunoassays, which were applied to the specific detection of the BSA protein.

Our work provides insights into CsPbX_3_ phase transformations in the presence of TEOS, water, and ammonia, thereby improving the fundamental understanding of the underlying silica growth chemistry and informing future synthetic and post-synthetic efforts toward the design of robust and efficient MHP@SiO_2_ core–shell nanoparticles with the desired surface functionality and with potential to work as fluorescent labels for bioimaging, biosensing, and molecular detection.

## Supporting Information Available

Experimental section; description of as-synthesized green emitting CsPbBr_3_ NCs optical and structural features; TEM images of CsPbBr_3_ NCs treated with different amounts TEOS and aqueous ammonia and control samples; elemental mapping images of samples B and G; evolution of PL intensity of core–shell nanoparticles in water solvent and change in relative fluorescence intensity as a function of time; HR-TEM image of CsPb_2_Br_5_@SiO_2_ NCs, table summary of structural features and reactant concentrations for the different synthesized SiO_2_-overcoated perovskite NPs; element quantification obtained from ICP–MS; and summary of the state of the art for the synthesis of MHP@SiO_2_ core–shell nanoparticles (PDF)
