# Supplementary material for: Monodispersed CsPb2Br5@SiO2 Core–Shell Nanoparticles as Luminescent Labels for Biosensing
Source: ACS Appl Nano Mater. 2021 Feb 12;4(2):2011–8. doi: 10.1021/acsanm.0c03340 (PMC8721593; doi:10.1021/acsanm.0c03340)
Supplement: Supplementary file 1 — an0c03340_si_001.pdf [file an0c03340_si_001.pdf]

Supporting Information

Monodispersed $CsPb_2Br_5@SiO_2$ Core-Shell Nanoparticles as Luminescent Labels for Biosensing

Cynthia Collantes,[†] Victoria González-Pedro,[*,†] María-José Bañuls[†,‡] and Ángel Maquieira[†,‡]

[†] IDM, Instituto Interuniversitario de Reconocimiento Molecular y Desarrollo Tecnológico, Departamento de Química, Universitat Politècnica de València. E-mail: vigonpe@upvnet.upv.es

[‡]Departamento de Química, Universitat Politècnica de València, Camino de Vera s/n, E46022 València, Spain.

Methods

Preparation of Cs-oleate. Cesium carbonate (Cs_2CO_3, 205 mg) was loaded into a 50 mL 2-neck flask and dried under vacuum for 10 min at room temperature. Octadecene (ODE, 10 mL) and oleic acid (OA, 0.620 mL) were injected under Ar into the flask and the temperature was raised to 120 °C under vacuum and mildly magnetic stirring for 40 min. Once Cs_2CO_3 was completely dissolved, the temperature was raised to 150 °C under Ar. Then the reaction was cooled down at room temperature.

Synthesis of $CsPbBr_3$. The synthesis of $CsPbBr_3$ NPs is based on the method described by Protesescu, L. *et al*. Briefly, $PbBr_2$ (138 mg), ODE (10 mL) and dried OA (1 mL) were loaded into a 100 mL 3-neck flask and degassed under vacuum at 120 °C for 45 min. Oleylamine (OAm, 1 mL) was then injected under Ar and the temperature was increased to 170 °C. At 165 °C, Cs-oleate was swiftly injected into the mixture. After 10 s, the reaction was cooled down in an ice-bath to stop crystal growth.

Synthesis of $CsPb_2Br_5@SiO_2$. In order to isolate $CsPbBr_3$ NCs, toluene (25 mL) was added into the flask and the solution centrifuged at 8,000 rpm for 10 min. The sediment was redispersed in toluene (25 mL) and centrifuged at 12,000 rpm for 10 min. The supernatant containing purified $CsPbBr_3$ NCs was collected in a bottle.

To obtain silica-coated NPs, 2 µL of concentrated aqueous ammonia and different volumes of TEOS and water were added into 0.5 mL of $CsPbBr_3$ NCs (40 nM in toluene)

under magnetic stirring at minimum speed and incubated overnight at room temperature in sealed 5 mL vials covered with Al foil to minimize exposure to light.

To collect the resulting core-shell nanoparticles, we applied sonication for 15 min, centrifuged at 9,000 rpm for 5 min, redispersed the sediment in 1 mL of toluene and sonicated again for 30 min.

To obtain functionalized nanoparticles, SiO_2 shell growth was terminated by adding 20 µL of (3-aminopropyl) triethoxysilane (APTES) or triethoxyvinylsilane (VTES). After washing and removal of excess of reagents, to gain information about the ligands covering the surface of the particles, ATR-FTIR spectroscopy was carried out on samples of pure alkoxysilanes and functionalized nanoparticles.

Immunoassay. *(i) Preparation of perovskite-antibody conjugates.* The anti- Bovine Serum Albumin antibody (antiBSA IgG) produced in rabbit (Sigma-Aldrich SAB4301142) were immobilized on the surface of MHP core-shell NPs via passive adsorption mechanism. For this purpose, 5 mg of MHP NCs were dispersed in 1 mL of increasing concentrations of antiBSA (0 to 100 µgmL^{-1}) and incubated under gentle mixing overnight at 4 ^{o}C. *(ii) Direct immunoassay.* BSA protein was immobilized onto the walls of polycarbonate vessels by their incubation overnight at 4 ^{o}C with 500 µgmL^{-1} (1 mL) of BSA in buffer saline phosphate (pH = 7). Then the vessels were rinsed with PBS-T and water and incubated for 2 h with the antibody-nanoparticle conjugate, which specifically recognizes protein. Finally, vessels where rinsed with PBS-T and water and luminescence was recorded by means of the spectrofluorometer equipment.

Characterization methods. UV-vis absorption spectra were recorded in a range of 300-800 nm by using a UV-visible spectrophotometer (Agilent 8453, Agilent Technologies). The photoluminescence spectra were obtained by a spectrofluorometer (PTI QMA4, Horiba) and PLQY was calculated by taking Rhodamine 6G dissolved in ethanol (PLQY = 0.95) or Quinine Sulfate in H_2SO_4 (PLQY = 0.6), depending on the emission wavelength. TEM images were taken by a transmission electron microscope 100 kV (JEM-1010, JEOL) and a field emission transmission electron microscope 200 kV with X-Ray detector (JEM 2100F, JEOL). Infrared spectra were recorded with an Attenuated total reflectance Fourier-transform infrared spectrometer in the range from 4000 to 600 cm^{-1} (ATR-FTIR

Tensor-27, Bruker). The Inductively coupled plasma mass spectrometry (ICP-MS) equipment used is an Agilent 7900, equipped with a Micromist-type concentric nebulizer, Scott-type spray chamber, nickel interface cones, off-axis double lens system and hyperbolic quadrupole as mass filter. For sample preparation, 1 mg of solid NPs has been brought to a final volume of 5 mL with ultrapure water. It has been digested in a Milestone ETHOS EASY high-pressure microwave oven at a maximum temperature of 220 ° C. An acid digestion with a mixture of HNO_3: HF (5:1) was underwent for the determination of Cs and Pb, and basic digestion with tetraethylammonium hydroxide (TEAH) for bromide detection.

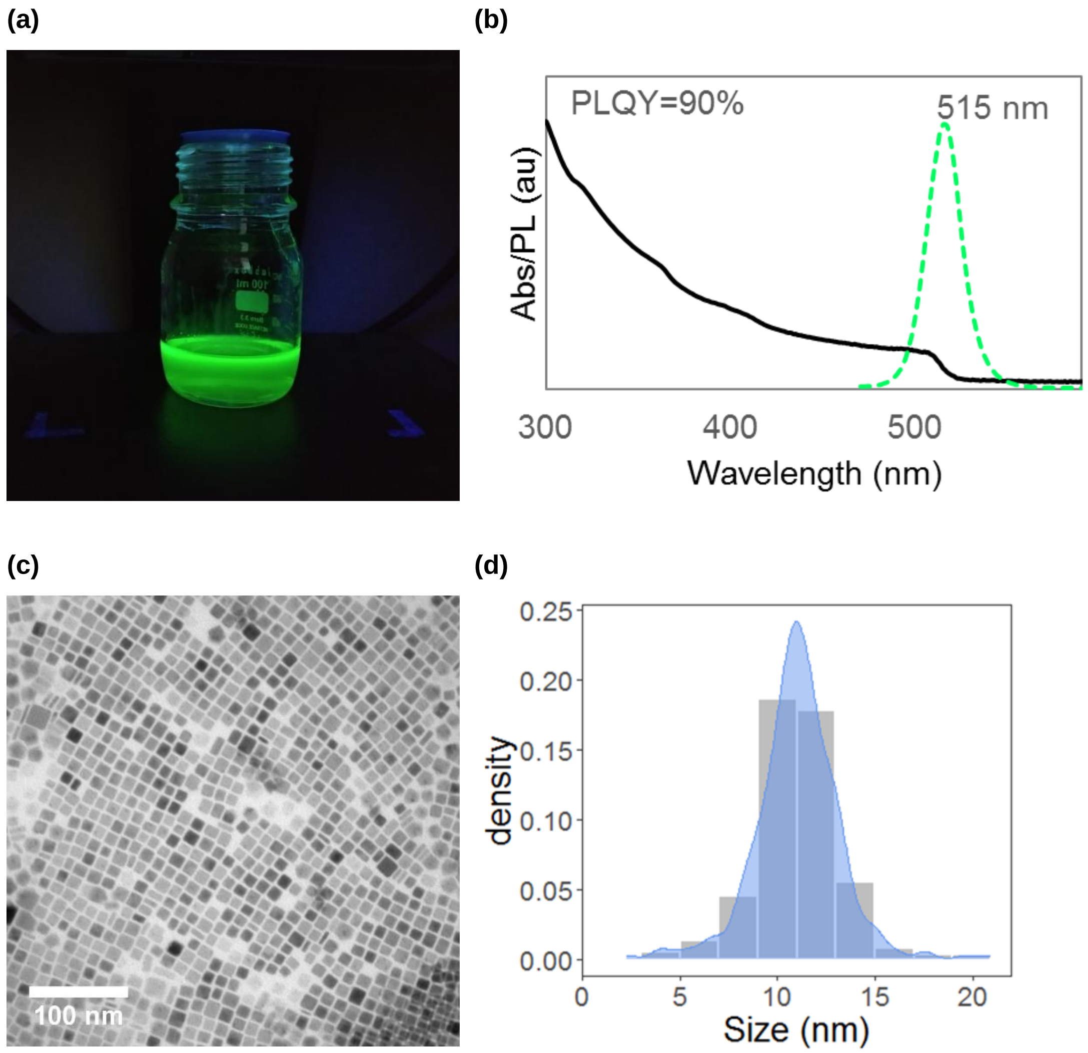

Figure. S1. (a) Picture of green emitting $CsPbBr_3$ NCs (λ_{exc} = 254 nm). (b) Absorbance (black line) and photoluminescence emission spectra (green line) for bare $CsPbBr_3$ NCs. The PL spectrum was recorded at excitation wavelength λ_{exc}= 480 nm. (c) Transmission electron microscopy image of cubic $CsPbBr_3$ perovskite NCs. (d) Particle Size distribution of $CsPbBr_3$ NCs with an average edge length of 11.04 ± 2.19.

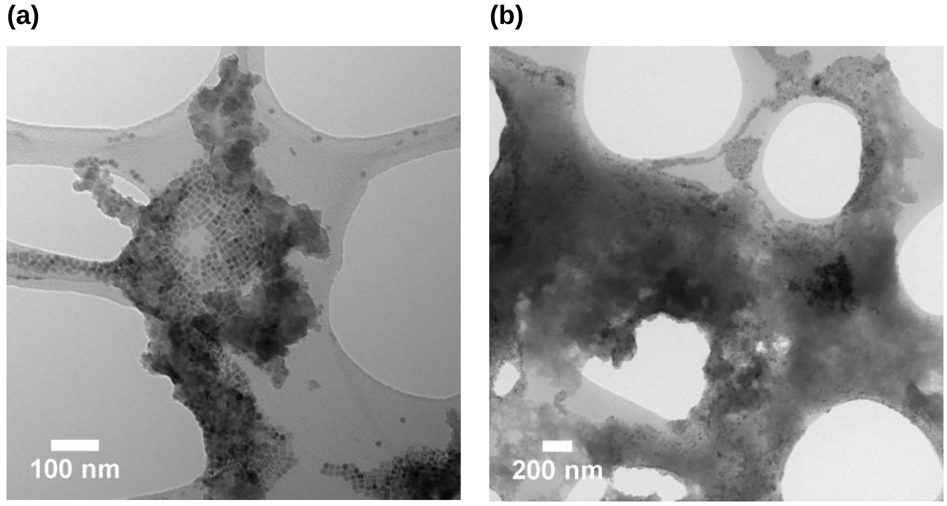

Figure S2 (a) TEM image of $CsPbBr_3$ NCs treated with 1440 µL of TEOS and 2 µL of aqueous ammonia, which led to an agglomeration of nanocrystals inside macroscale alkoxysilane composite. (b) Control of sample B, treated with 310 µL of TEOS in the absence of aqueous ammonia.

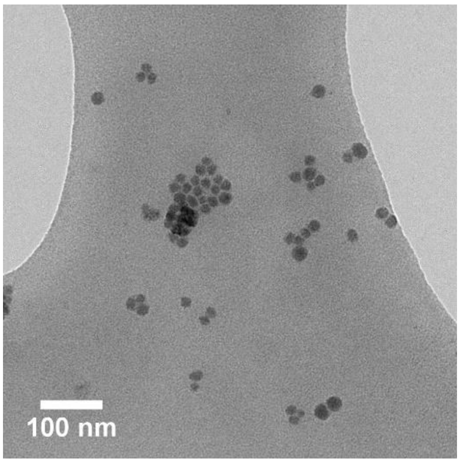

Figure S3. TEM image of MHP NCs treated with 310 µL of TEOS and 2 µL of aqueous ammonia and 90 µL of water, which led to the formation of core-free SiO_2 nanoparticles.

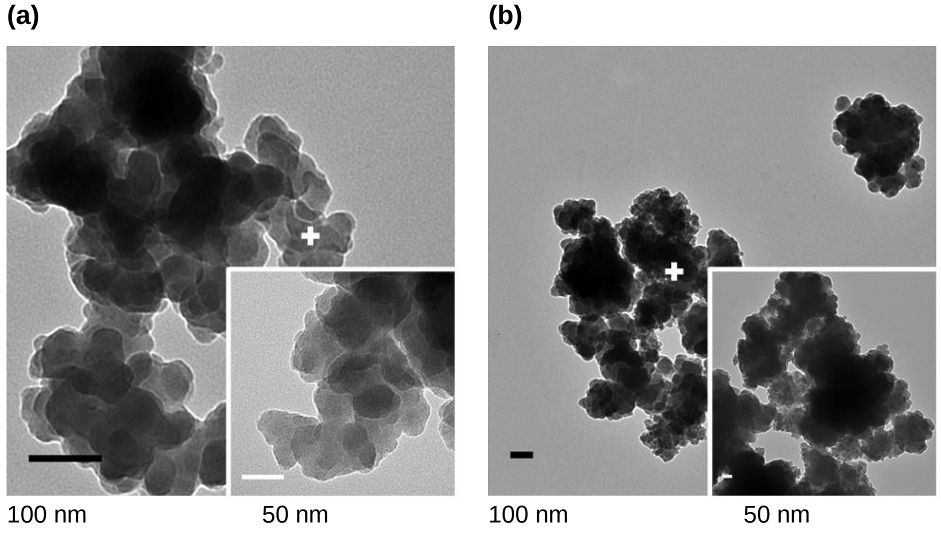

Figure S4. TEM image of MHP NCs treated with (a) 480 µL and (b) 960 µL of TEOS, 2 µL of aqueous ammonia 25% (w/w) and 28 µL of water, which led to macroscale silica agglomerates.

(a) 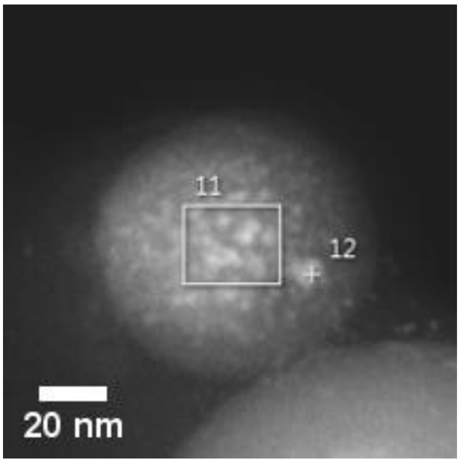 (b) 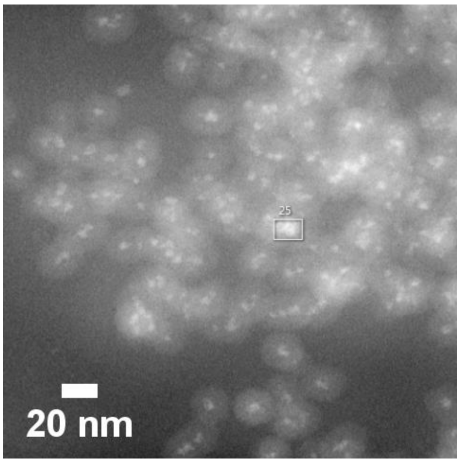

	Sample A			Sample B		
**Element**	**Wt%**	**Wt% σ**	**Atomic %**	**Wt%**	**Wt% σ**	**Atomic%**
O	50.48	0.46	65.34	49.63	3.18	63.67
Si	46.40	0.45	34.23	49.29	2.44	36.14
Br	0.52	0.12	0.13	0.44	0.39	0.11
Pb	2.60	0.27	0.26	0.63	1.07	0.06

Figure S5. Elemental mapping images of Sample B (a) and G (b) showing the atomic distribution of Si, O, Br and Pb of a core-shell NP of nm obtained by EDX TEM. From this data set it is noteworthy that the shells present a Si:O atomic ratio close to 1:2 in good agreement with the theoretical value. Regarding the elemental composition of small MHP nanoclusters of 2-3 nm in diameter trapped inside, only Pb and Br are detectable. This fact could be due to the ultrasmall size of these nanoparticles, which are damaged by electron irradiation and disappear after the spectra acquisition. Hence, the contribution of Cs is too small or too short-lived to be detected. In addition, the elemental analysis has not shown accurate Pb:Br stoichiometry, which could be attributed to the lack of reliability for EDX quantification when the particles are too small and the thickness of the shell is remarkable. [1,2]

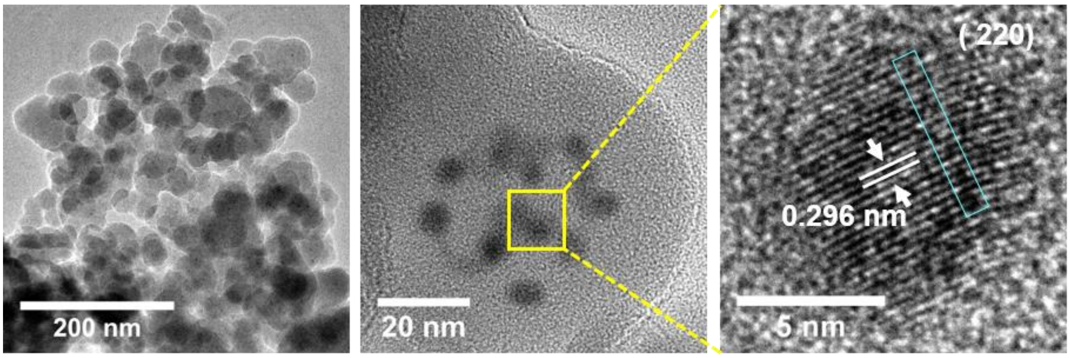

Figure S6. High-resolution TEM image of MHP NCs treated with 310 µL of TEOS, 2 µL of aqueous ammonia 25% (w/w) and 48 µL of water, which led to the formation of agglomerated perovskite core-shell NCs. The d-spacing analysis carried out on the HRTEM images of nanocrystals show a value of 0.3 nm, matching with the value of the (220) plane of the tetragonal structure of $CsPb_2Br_5$.

(a) (b)

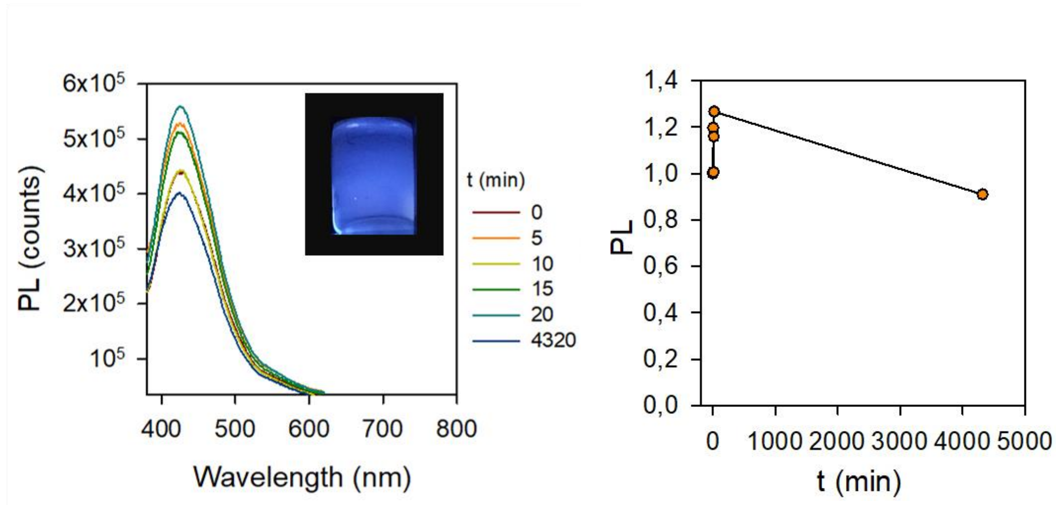

Figure S7. (a) Evolution of PL intensity of core-shell nanoparticles in water solvent and (b) change in relative fluorescence intensity as a function of time. The inset in figure (a) depicts a picture of blue emitting core-shell NPs in water (λ_{exc} = 254 nm).

Table S1. Experimental parameters for synthesizing SiO_2-overcoated perovskite nanoparticles

Sample	***$V_{[NH4OH]}$ (25%.) (μL)***	***V_{TEOS} (μL)***	***Additional Water Volume (μL)***	***Fractionated Addittion TEOS (μLmin^{-1})***	***Fractionated Addittion NH_4OH (μLmin^{-1})***	***Particle Morphology***	***Particle size (nm)***	***Nº core /particle***	***Particle distribution***	***Population percentage (%)***
A	2	60	0	--	--	Elipsoidal	d_A = 35.5 ± 8.4<br>d_B = 20. ± 3.4	1-15	Partially aggregated	~98.5
						Spherical	d =162 ± 38	--	Isolated	< 1
						Nanowires	Width = 56 ± 14<br>Length = variable	--	Isolated	<0.5
B	2	310	0	--	--	Spherical	d = 101.4 ± 6.5	>10	Aggregated	~80
						Spherical	d = 40.9 ± 4.2	1-5	Aggregated	~20
C	2	480	0	--	--	Spherical	d =19.7 ± 2.4	1-5	Aggregated	>99
D	2	960	0	--	--	Spherical	d = 33.8 ± 4.0	1-5	Aggregated	>99
E	2	310	0	0.74	0.1	Spherical	d = 99.5 ± 32.1	N.D.	Agglomerated	>99
F	2	310	28	0.74	0.1	Spherical	d = 33.9 ± 2.1	5-13	Monodispersed	>99
G	2	310	48	0.74	0.1	Spherical	d = 32.4 ± 1.2	1-3	Monodispersed	>99

Table S2. Particle size analysis of MHP@SiO_2

	Mean	*Std*
Particle size (nm)	*36.1*	*4.5*
Shell thickness (nm)	*13.3*	*1.8*
Core size (nm)	*2.0*	*0.8*
Cores/particle	*2.8*	*1.1*

Table S3. Element quantification obtained from Inductively coupled plasma mass spectrometry (ICP-MS).

Element	*(mg/g)*	*(mmol/g)*
**Br (mg/g)**	44.50 ± 0.40	0.557 ± 0.005
**Cs (mg/g)**	3.02 ± 0.04	0.0227 ± 0.0003
**Pb (mg/g)**	8.39 ± 0.05	0.0405 ± 0.0002

Table S4. Summary of the state of art for the synthesis of MHP@SiO_2 core-shell nanoparticles.

Core-shell	*Methodology*	*PLQY in aprotic solvent (%)*	*Particle size (nm)*	*Morphology/Monodispersion*	*PLQY in protic solvent (%)*	*Stability in protic medium*	*Application*	*Ref.*
$CsPbBr_3/SiO_2$	Room-temperature crystal growth inside pre-synthesized amine functionalized silica micelles.	NR	5.2	Quasi spherical/Monodisperse	61.2 (ethanol)	34 days (ethanol) (PLQY~38%)	NR	3
$CsPbBr_3/SiO_2$	Adapted reverse microemulsion method	84	120	Hexagonal/Monodisperse	82%	4h (40% left) in water	White-LED Cell imaging	4
$CsPbBr_3/SiO_2$	Modified supersaturated recrystallization method.	90 (toluene)	25-30	Quasi spherical/Monodisperse	NR	40 min of ultrasonication in water (0% left)	NR	5
$CsPbX_3/SiO_2$ Janus NPs	Combination of water-triggered transformation process and a sol–gel method	80-85 (hexane)	11.2 ($CsPbBr_3$) 3.4-12.6 (SiO2)	Janus NCs/Monodisperse	NR	7 days in water:hexane (50:50) (20% left)	White-LED devices	6
$CsPbBr_3/SiO_2$	Modified hot injection method, wherein certain part of oleylamine was substituted with APTES	NR	14-19.4	Cubes/ Monodisperse	NR	48 h in water (not reported value)	Cell imaging	7

Continuation of Table S3

$CsPbBr_3/SiO_2$	Adapted sol-gel process	78 (toluene)	170	Spherical/Monodisperse	NR	20 days in water (50% left)	A frequency up-converted laser device	8
$MAPbBr_3@SiO_2$	Adapted sol-gel process	89 (toluene)	150-500 nm	Spherical/NR	NR	7 days 80% relative humidity (20% left)	NR	9
$CsPbBr_3/SiO_2$	In situ growth in mesoporous templates	48 (powder)	200-1000 nm	Spherical/ NR	NR	NR	NR	10
($MAPbBr_3@SiO_2$/ poly (vinylidene fluoride)	In situ growth into hollow siliceous nanospheres	85.5 (powder)	20.7	Spherical/ Monodisperse	NR	2 h in water (40% left)	White-LED	11
$CsPbBr_3/SiO_2$	Adapted sol gel processes	87 (DMF)	< 125	Spherical/Polydisperse	NR	NR	NR	12
$CsPbBr_3/SiO_2$	Water triggered transformation and sol-gel method	NR	Macroscale NPs	Not applicable	NR	24 h in water (20% left)	White-LED	13
$CsPbBr_3/SiO_2$	Sol-gel process assisted with water traces	78	Macroscale NPs	Not applicable	NR	3 months in air (0% left)	White-LED	14
$CsPb_2Br_5/SiO_2$	Adapted sol-gel process	90 (toluene)	32.4	Spherical/Monodisperse	5	3 days in water (10% left)	Protein biosensing	This work

NR. Not reported

(1) Vidick, D.; Ke, X.; Devillers, M.; Poleunis, C.; Delcorte, A.; Moggi, P.; Van Tendeloo, G.; Hermans, S. Heterometal Nanoparticles from Ru-Based Molecular Clusters Covalently Anchored onto Functionalized Carbon Nanotubes and Nanofibers. *Beilstein J. Nanotechnol.* **2015**, *6* (1), 1287–1297. https://doi.org/10.3762/bjnano.6.133.

(2) Shamsi, J.; Urban, A. S.; Imran, M.; De Trizio, L.; Manna, L. Metal Halide Perovskite Nanocrystals: Synthesis, Post-Synthesis Modifications, and Their Optical Properties. *Chem. Rev.* **2019**, *119* (5), 3296–3348. https://doi.org/10.1021/acs.chemrev.8b00644.

(3) Cheng, J.; Yuan, S.; Zhu, L.; Chen, L.; Liu, C.; Tong, H.; Zeng, H. Room-Temperature in Situ Synthesis of Highly Efficient $CsPbBr_3/SiO_2$ Sol in Entirely Ethanol Solvent by Constructing Amine-Functionalized Silica Micelles. *Langmuir* **2020**, *36* (13), 3565–3572. https://doi.org/10.1021/acs.langmuir.0c00108.

(4) Ding, N.; Zhou, D.; Sun, X.; Xu, W.; Xu, H.; Pan, G.; Li, D.; Zhang, S.; Dong, B.; Song, H. Highly Stable and Water-Soluble Monodisperse $CsPbX_3/SiO_2$ Nanocomposites for White-LED and Cells Imaging. *Nanotechnology* **2018**, *29* (34), 345703. https://doi.org/10.1088/1361-6528/AAC84D.

(5) Zhong, Q.; Cao, M.; Hu, H.; Yang, D.; Chen, M.; Li, P.; Wu, L.; Zhang, Q. One-Pot Synthesis of Highly Stable $CsPbBr_3@SiO_2$ Core-Shell Nanoparticles. *ACS Nano* **2018**, *12* (8), 8579–8587. https://doi.org/10.1021/acsnano.8b04209.

(6) Hu, H.; Wu, L.; Tan, Y.; Zhong, Q.; Chen, M.; Qiu, Y.; Yang, D.; Sun, B.; Zhang, Q.; Yin, Y. Interfacial Synthesis of Highly Stable $CsPbX_3$/Oxide Janus Nanoparticles. *J. Am. Chem. Soc.* **2018**, *140* (1), 406–412. https://doi.org/10.1021/jacs.7b11003.

(7) Song, W.; Wang, Y.; Wang, B.; Yao, Y.; Wang, W.; Wu, J.; Shen, Q.; Luo, W.; Zou, Z. Super Stable $CsPbBr_3@SiO_2$ Tumor Imaging Reagent by Stress-Response Encapsulation. *Nano Res.* **2020**, *13* (3), 795–801. https://doi.org/10.1007/s12274-020-2697-9.

(8) Li, S.; Lei, D.; Ren, W.; Guo, X.; Wu, S.; Zhu, Y.; Rogach, A. L.; Chhowalla, M.; Jen, A. K. Y. Water-Resistant Perovskite Nanodots Enable Robust Two-Photon Lasing in Aqueous Environment. *Nat. Commun.* **2020**, *11* (1), 1–8. https://doi.org/10.1038/s41467-020-15016-2.

(9) Huang, S.; Li, Z.; Kong, L.; Zhu, N.; Shan, A.; Li, L. Enhancing the Stability of

CH3NH3PbBr3 Quantum Dots by Embedding in Silica Spheres Derived from Tetramethyl Orthosilicate in "Waterless" Toluene. *J. Am. Chem. Soc.* **2016**, *138* (18), 5749–5752. https://doi.org/10.1021/jacs.5b13101.

(10) Dirin, D. N.; Protesescu, L.; Trummer, D.; Kochetygov, I. V.; Yakunin, S.; Krumeich, F.; Stadie, N. P.; Kovalenko, M. V. Harnessing Defect-Tolerance at the Nanoscale: Highly Luminescent Lead Halide Perovskite Nanocrystals in Mesoporous Silica Matrixes. *Nano Lett.* **2016**, *16* (9), 5866–5874. https://doi.org/10.1021/acs.nanolett.6b02688.

(11) Huang, Y.; Li, F.; Qiu, L.; Lin, F.; Lai, Z.; Wang, S.; Lin, L.; Zhu, Y.; Wang, Y.; Jiang, Y.; Chen, X. Enhancing the Stability of CH $_3$ NH $_3$ PbBr $_3$ Nanoparticles Using Double Hydrophobic Shells of SiO_2 and Poly(Vinylidene Fluoride). **2019,** *11* (29), 26384-26391. https://doi.org/10.1021/acsami.9b07841.

(12) Cai, J.; Gu, K.; Zhu, Y.; Zhu, J.; Wang, Y.; Shen, J.; Trinchi, A.; Li, C.; Wei, G. Highly Stable $CsPbBr_3$@SiO_2 Nanocomposites Prepared via Confined Condensation for Use as a Luminescent Ink. *Chem. Commun.* **2018**, *54* (58), 8064–8067. https://doi.org/10.1039/c8cc04130j.

(13) Liu, Y.; Li, F.; Liu, Q.; Xia, Z. Synergetic Effect of Postsynthetic Water Treatment on the Enhanced Photoluminescence and Stability of $CsPbX_3$ (X = Cl, Br, I) Perovskite Nanocrystals. *Chem. Mater.* **2018**, *30* (19), 6922–6929. https://doi.org/10.1021/acs.chemmater.8b03330.

(14) Sun, C.; Zhang, Y.; Ruan, C.; Yin, C.; Wang, X.; Wang, Y.; Yu, W. W. Efficient and Stable White LEDs with Silica-Coated Inorganic Perovskite Quantum Dots. *Adv. Mater.* **2016**, *28* (45), 10088–10094. https://doi.org/10.1002/adma.201603081.
